# Rectus Femoris Muscle and Phase Angle as Prognostic Factor for 12-Month Mortality in a Longitudinal Cohort of Patients with Cancer (AnyVida Trial)

**DOI:** 10.3390/nu15030522

**Published:** 2023-01-19

**Authors:** Cristina García-García, Isabel María Vegas-Aguilar, Rosalía Rioja-Vázquez, Isabel Cornejo-Pareja, Francisco J. Tinahones, José Manuel García-Almeida

**Affiliations:** 1 Facultad de Medicina, Universidad of Málaga, 29010 Málaga, Spain; 2 Unidad de Gestión Clínica en Endocrinología y Nutrición, Hospital Universitario Virgen de la Victoria, 29010 Málaga, Spain; 3 Instituto de Investigación Biomédica de Málaga (IBIMA), Hospital Universitario Virgen de la Victoria, 29010 Málaga, Spain; 4 Asociación de Enfermeras de Nutrición y Dietética (ADENYD), 14004 Córdoba, Spain; 5 Centro de Investigacion Biomedica en Red de la Fisiopatología de la Obesidad y Nutricion (CIBEROBN), Instituto de Salud Carlos III (ISCIII), 29010 Malaga, Spain; 6 Servicio de Endocrinología y Nutrición, Quirónsalud Hospital, 29010 Málaga, Spain

**Keywords:** cancer, disease-related malnutrition, bioelectrical impedance, phase angle, rectus femoris muscle, mortality

## Abstract

Background: Cancer-related malnutrition is still unrecognized and undertreated in clinical practice. The morphofunctional assessment of disease-related malnutrition (DRM) is a new approach that focuses on evaluating changes in body composition and function. The aim of this study is to evaluate the prognostic value of classic and emerging assessment of malnutrition at 12-months survival in cancer patients. Methods. We conducted a prospective study on cancer outpatients. Bioelectrical impedance with phase angle (PhA), nutritional ultrasound by rectus femoris cross-sectional area (RFCSA), hand grip strength, and “Timed Up and Go Test” (TUG) were evaluated as predictors of mortality. Results. Fifty-seven patients were included. The non-survivors had lower PhA values than the survivors (4.7° vs. 5.4°; *p* < 0.001), and we had the same results with RFCSA 2.98 cm^2^/m^2^ vs. 4.27 cm^2^/m^2^ (*p* = 0.03). Cut-off points were identified using the ROC (receiver operating characteristic) curves for PhA (≤5.6° cancer patients, ≤5.9° men, ≤5.3° women), RFCSA (≤4.47 cm^2^/m^2^ cancer patients, ≤4.47° men, ≤2.73° women) and rectus femoris-Y-axis (RF-Y-axis; ≤1.3 cm cancer patients, ≤1.06 men, ≤1 women). In multivariate logistic regression analysis, we found that high PhA was significantly associated with a lower mortality hazard ratio (HR: 0.42 95% CI: 0.21–0.84, *p* = 0.014). Likewise, high RFCSA was associated with a decrease in mortality risk in the crude model (HR: 0.61 95% CI: 0.39–0.96, *p* = 0.031). This trend was also maintained in the adjusted models by the confounding variables. Conclusions. Low PhA and RFCSA values are significant independent predictors of mortality in cancer patients. These cut-off points are clinical data that can be used for nutritional assessment and the prediction of clinical outcomes.

## 1. Introduction

Cancer diseases are the second leading cause of death worldwide. Disease-related malnutrition (DRM) refers to an imbalance between nutrient and energy intake and nutrient and energy requirements, which leads to metabolic and functional changes in nutritional status and body composition markers [1,2]. It is estimated that 10–20% of cancer patients die due to the consequences of malnutrition rather than the tumor itself [1]. Cancer-related malnutrition is still unrecognized and undertreated in clinical practice. Immediately after cancer is diagnosed, nutritional guidelines suggest screening cancer patients for nutritional risk, and if present, a complete nutritional assessment should be performed [2].

Patients with cancer experience changes in their body composition and muscle function along with the disease process. Cachexia and sarcopenia are highly prevalent in this type of patient. Cancer cachexia consists of systemic inflammation, involuntary loss of lean body mass, with or without loss of adipose tissue, and negative protein balance [3,4,5]. Sarcopenia is considered a muscle disease that causes low muscle strength and is associated with low muscle quantity and quality [4].

The diagnosis of DRM could be applied to the recent Global Leadership Initiative on Malnutrition (GLIM) criteria, contributing to measuring some different etiologic and phenotypic criteria [6]. In 1744 patients with cancer in a multicenter cohort study, Xi Zhang et al. showed that the FFMI score was associated with all-cause mortality ((HR): 0.72; *p* < 0.001) in men and ((HR): 0.88; *p* = 0.048) in women patients with cancer. These results highlight the usefulness of the FFMI for routine clinical assessment and prognostic estimation in patients with cancer [7].

There are multiple limitations in the classic parameters for nutrition assessment, including body mass index (BMI), weight loss, food intake, or standard laboratory parameters such as albumin or lymphocytes. Because of this, a new approach to nutrition focused on the assessment of nutritional status by evaluating changes in composition and function using parameters such as phase angle (PhA) and other electrical measurements of bioelectrical impedance analysis (BIA), hand grip strength (HGS), functional tests, nutritional ultrasound (NU®), or laboratory parameters such as C-reactive protein (CRP)/prealbumin [8] is necessary. The new concept of morphofunctional assessment of DRM consists of integrating the classic parameters for nutrition assessment with emerging nutritional techniques which determine the function and body composition. 

The life expectancy of patients with advanced cancer can be several months or several years. Skeletal muscle loss is a defining feature of cancer cachexia. It directly affects health-related quality of life (HRQoL) and survival. Sarcopenia also negatively affects physical function and HRQoL [1,4,9,10]. A recent meta-analysis shows that low skeletal muscle mass is an essential predictor of treatment toxicity in oncologic patients [11].

Nutritional therapies may include dietary counseling, with or without oral nutrition supplements, enteral nutrition, or parenteral nutrition; the choice depends on the patient’s current situation. The effectiveness of oral nutrition supplements for improving nutritional status and QOL has been provided, and adherence to nutritional therapy is rarely addressed. Health outcomes can be strongly affected by monitoring and optimizing nutritional treatment, which increases the nutrition intervention benefits [12]. 

DRM is a multidimensional problem that needs a multimodal approach [13]. Our aim was to evaluate the prognostic value of classic (weight loss, body mass index, reduction intake) and emerging assessment of malnutrition (phase angle, body cell mass, rectus femoris cross-sectional area, hand grip strength, and Timed Up and Go Test), adherence, and HRQoL on 1-year survival in cancer patients. These results can contribute to the improvement of clinical practice to generate real-world evidence.

## 2. Materials and Methods

### 2.1. Study Design and Population

In a prospective and longitudinal cohort study, cancer outpatients were referred to the Department of Endocrinology and Nutrition at a tertiary-level hospital during the study period (November 2018—November 2021). Inclusion criteria were patients older than 18 years diagnosed with neoplasm (without distinguishing cause of pathology or age) and DRM who received active treatment. The exclusion criteria were estimated contraindications to BIA, ECOG (Eastern Cooperative Oncology Group) >2, severe chronic kidney disease or hepatic failure, insulin-treated diabetes, and failure to provide signed informed consent.

The study was approved by the Clinical Research Ethics Committee of The Virgen de la Victoria University Hospital (number 0358-N-18, approval date 22 March 2018) and was performed in compliance with the ethical and legal standards required for biomedical research according to the Declaration of Helsinki. The aim of the study was individually explained to all patients, with written information given. Both written and verbal informed consent were requested. We tested the hypothesis that PhA was an independent predictor of 12-month mortality in cancer patients. We calculated the sample size using the findings of Gupta et al. [14] in a population with advanced colorectal cancer. The analysis outcome was PhA ≤ 5.57° [RR (relative risk) = 10.75 (CI (confidence interval) 95% 1.92–60.24); *p* = 0.007. Cut-off point: ≤5.6°. 24/52 of events/total participants]. Thus, at an alpha error of 0.05, a power of 80%, and a loss rate of 10%, a minimum of 55 patients were needed to attain sufficient power. 

### 2.2. Morphofunctional Assessment of Disease-Related Malnutrition

**Figure 1:**
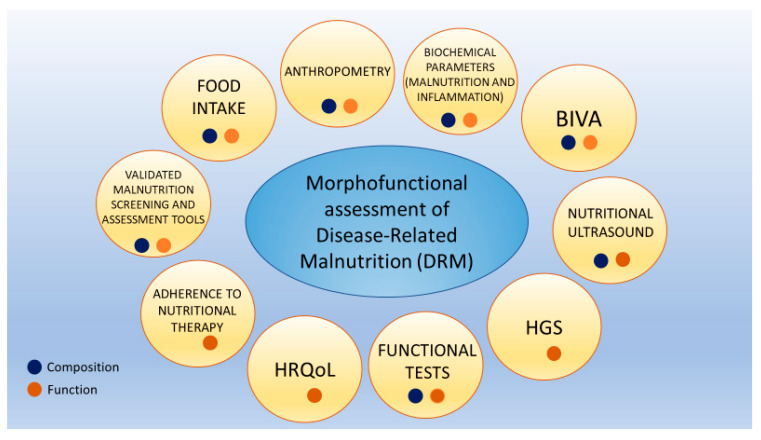
Morphofunctional assessment of disease-related malnutrition. Each assessment area can provide insights into body composition, body function, or both. Abbreviations. HRQoL: health-related quality of life; HGS: hand grip strength; BIVA: bioelectrical impedance vector analysis). To interpret the color references in this figure legend, please see the Web version of this article. Source: own elaboration.

Morphofunctional assessment of DRM consists of integrating different classical and emerging nutritional assessment techniques. This focuses on nutritional status assessment through the evaluation of composition and function changes. This is performed by utilizing parameters such as PhA, BCM, and other bioimpedance electrical measurements of bioimpedance, nutritional ultrasound, HGS, functional tests, biochemical parameters such as CRP/prealbumin, HRQoL, and monitoring adherence to nutritional therapy (Figure 1) [8].

#### 2.2.1. Clinical, Anthropometric, and Nutritional Data

We collected the tumor type, clinical staging, TNM cancer staging system, oncology therapy, surgery, ECOG, and other clinical information by interview or medical record. The patient’s body mass index (BMI) was determined according to the World Health Organization. 

The diagnosis and classification of malnutrition were carried out using different tests, including the Nutriscore tool [15], subjective global assessment (SGA) [16], mini nutritional assessment full form (MNA®-FF) [17], and GLIM criteria [6] which stipulate that a minimum of one etiologic and one phenotypic criterion must be present at the same time. The following variations were assessed for phenotypic GLIM criteria: (1) unintentional weight loss >5% within the last 6 months; (2) BMI < 20 kg/m2 (if <70 years) or <22 kg/m^2^ (if >70 years); and (3) reduction of muscle mass based on appendicular skeletal muscle index (ASMI), fat-free mass index (FFMI), and appendicular lean mass (ALM) measured by BIA. We also assessed HGS, mid-arm circumference (MAC), and arm muscular circumference (AMC). The following anthropometric measures were gathered: BMI, weight, and height. Height was calculated at baseline with a stadiometer (Holtain Limited, Crymych, UK), and weight was calculated with a weighting scale adjusted to 0.1 kg (SECA 665, Hamburg, Germany). The FFMI cut-off was <17 kg/m^2^ for men or <15 kg/m^2^ for women, according to The European Society for Clinical Nutrition and Metabolism (ESPEN) [18]. The ASMI cut-off was <7 kg/m^2^ for men or <5.5 kg/m^2^ for women, according to the European Working Group on Sarcopenia in Older People [4]. The ALM cut-off was <21.4 for men and 14.1 for women [6]. MAC was measured using flexible, non-elastic tape. This value and triceps skinfold were used to estimate AMC. Low muscle mass contemplates a value below the fifth percentile (p5) [19].

Calf circumference (CC), one anthropometric approach gaining interest as a marker of muscle mass, was measured with an inelastic and flexible measuring tape in the gastrocnemius muscle. The CC cut-off was <34 cm for men and <33 cm for women (BMI = 18.5–24.9 kg/m^2^, for other BMIs, adjustment factors) [20]. We considered that all participants met the GLIM etiologic criteria for chronic disease-related due to cancer. The food intake count was measured by quartiles of the previous 5 days (0–25% almost nothing, 25–50% less than half of usual, 50–75% more than half of usual, 75–100% almost normal). This method is recommended by ESPEN in the “Nutrition Day” [21,22]. Food intake was measured using a nutritional assessment that incorporates questions related to intake (MNA®-FF and SGA). 

#### 2.2.2. Bioelectrical Impedance Analysis Assessment

The impedance measurements were performed with a phase-sensitive single-frequency analyzer (Nutrilab, Akern® Srl, Pontassieve, Italy) which applies an alternating sinusoidal electric current of 400 µA at 50 kHz (±0.1%; resolution Rz: ±1%, Xc: ±1%, coefficients of variation (CV) <2%). Standard whole-body tetrapolar measurements were performed according to the manufacturer’s guidelines [23]. The position was supine with a leg opening of 45° compared to the median line of the body, and the upper limbs positioned 30° away from the trunk. After cleansing the skin with isopropyl alcohol, one Ag/AgCl very low-impedance electrode (BIVAtrodes, Akern® Srl, Italy) was placed on the back of the right hand and one electrode on the corresponding foot; each electrode included a sensor and injector area separated by a distance of 5 cm according to standard protocol [24]. To avoid disturbances in fluid distribution, the subject was instructed to abstain from food and drink for > 2 h before the test [25]. Resistance (R, Ω), reactance (Xc, Ω), PhA (°), standardized phase angle (SPhA, °), body cell mass (BCM, kg), FFMI (kg/m^2^), fat mass index (FMI, kg/m^2^), ALM (kg), appendicular skeletal muscle index (ASMI, kg/m^2^), and hydration status (%) were collected.

Due to the movement of fluid upon changing from standing to recumbency which affects values R and Z directly, five minutes were spent in a supine position before BI measurements were taken. Bioelectrical impedance vector analysis (BIVA) was performed using the RXc graph to classify hydration status and nutritional status [26]. BIA measurements of patients were standardized for sex and age using data from healthy Italian adults [27,28]. Euhydration is described as 72.7–74.3%, with over-hydration exceeding 74.3% þ 1 SD of euhydration and dehydration less than 72.7%, −1SD of euhydration [29,30,31]. The BIVA approach has garnered attention as a tool to monitor and assess patients’ nutrition status and hydration. This is essential to show the PhA and vector position’s independent roles when assessing hydration and nutrition status in a clinical context. Thus, it is appropriate to consider hydration as a possible confounder of interpretations of PhA in malnutritional assessment. BIVA uses the 50, 75, and 95% confidence ellipses of reference populations to classify individual and group vectors. The bioelectrical impedance vector distribution analysis shows a situation of inflammation and cellular injury associated with cancer.

#### 2.2.3. Nutritional Ultrasound®

**Figure 2:**
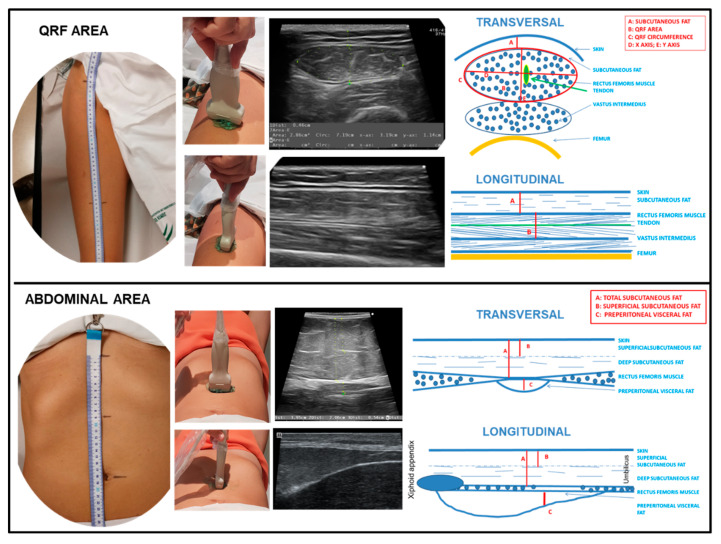
Transversal and longitudinal section of the rectus femoris (QRF) muscle and the abdominal area. Representation of the anatomical measurement area. Position of the ultrasound transducer. Image and scheme of the anatomical structures. To interpret the color references in this figure legend, please see the Web version of this article. Source: own elaboration.

Nutritional ultrasound® is a novel concept in which the body composition is assessed via ultrasound. It comprises two dimensions with the aim of assessing FFM (rectus femoris muscle ultrasound) and evaluating FM (abdominal adipose tissue ultrasound) [8,32,33,34,35,36]. We performed a thorough nutritional ultrasound® assessment with a HITACHI® ALOKA F37 ultrasound scanner with an Aloka UST-5413 Linear Array transducer with a frequency range of 5.0 MHz–10.0 MHz in B-mode in a transverse position (Hitachi® Europe, Ltd. Japan). The patient was relaxed and supine, lying with the knee fully extended. Scans for rectus femoris muscle ultrasound were carried out in the area two-thirds of the distance between the superior pole of the patella and the anterosuperior iliac spine. The measures taken were RFCSA, RF-circumference (RFC), RF-X-axis, and RF-Y-axis, which correspond to the linear measurement of the distance between the muscular limits of the rectus femoris (lateral and anteroposterior) and RF-adipose tissue [32]. Scans were performed for abdominal adipose tissue ultrasound at the midpoint between the xiphoid appendix and the navel on the midline. The cross-section of the anatomical structures is shown in Figure 2.

#### 2.2.4. Hand Grip Strength

A Jamar dynamometer (Asimow Engineering Co., Los Angeles, CA, USA) was used to measure HGS in the dominant hand. Patients were sitting with wrist and forearm in a neutral position, elbow bent to 90 degrees, forearm neutrally rotated, and shoulder adducted. The mean value was calculated by asking patients to complete three successive contractions spaced one minute apart [37]. 

#### 2.2.5. Functional Tests: Timed Up and Go Test

The Timed Up and Go Test (TUG) measures how long patients take to stand from a seated position, walk three meters, turn around, walk back, and sit again. The score refers to how many seconds were needed to finish the test activity. It has adequate validity, and fall risk can be predicted with above 80% specificity and sensitivity. The patient is completely independent if the score is less than 10 s, independent for main transfers if the score is less than 20 s, and requires assistance if the score is more than 30 s [38,39].

### 2.3. Biochemical Parameters (Malnutrition and Inflammation)

We measured specific biomolecular markers that assess nutrition and inflammation, such as prealbumin and C-reactive protein (CRP)/prealbumin ratio. Prealbumin is much more sensitive to any changes in whole-body protein status than albumin, and hydration status does not affect it [40]. Its association with CRP levels, a pure marker for inflammation in the body, may increase its interest as a predictor of morbidity and mortality and of nutritional/inflammatory changes [41,42]. CRP/prealbumin is independently correlated with hospital mortality [43].

### 2.4. HRQoL and Adherence

To assess the HRQoL, we used two specific tests: EORTC QLQ C30 and NutriQoL®. The QLQ-C30 contains 30 questions and 14 scales, each representing a particular symptom or aspect of function, plus 1 global quality of life scale [44]. The NutriQoL® questionnaire has 17 questions, each divided into 2 parts, regarding items related to home enteral nutrition and the patient’s perceived importance of that item [45]. 

Furthermore, two indirect methods to assess adherence to oral nutritional supplements were used. Daily self-reported consumption was used to quantify intake quartiles of each bottle, and a validated specific questionnaire was used to assess qualitative adherence aspects to oral nutritional supplements (Wanden-Bergue et al.) [46].

### 2.5. Follow-Up and Outcome Measures

The primary endpoint was to evaluate the prognosis value of morphofunctional assessment of the DRM for cancer outpatients (overall survival). Overall survival was defined as death occurring during the 12 months follow-up. The end of the follow-up was on 20 November 2021. The median follow-up period was 12.9 months (4.9–19.9). None of the patients were lost to follow-up. 

### 2.6. Statistical Analysis

The JAMOVI program (version 2.2.3.0) was principally used to carry out statistical analyses of the data. We characterized our patient cohort using descriptive statistics. The Shapiro–Wilk test was used to confirm the normality of the quantitative variable distribution. Descriptive statistics were used to analyze quantitative variables (mean and SD or median and interquartile range) and categorical variables (absolute and relative frequency). Student’s *t*-test or the Mann–Whitney U test were used to compare the clinical data and BIVA values between survivor and non-survivor patients.

The chi-squared (or Fisher’s exact test) was used to compare categorical variables. The relationship was also analyzed with Pearson or Spearman correlations models according to normal distribution. To confirm whether different variables are factors that can predict mortality, we conducted binary logistic regression analysis using an adjusted model in the presence or absence of death as a dependent variable. Prior to performing binary logistic regression in the adjustment model, we conducted a correlation analysis between the collected morphofunctional data and mortality; variables that had a significant correlation to others were used in the analysis. The Hosmer–Lemeshow test was performed to assess the goodness of fit in logistic regression analysis, and the model was considered a good fit with *p* < 0.05.

Evaluation of PhA, nutritional ultrasound®, and HGS diagnostic performance was based on the receiver operating characteristic (ROC) curve and the area under the curve (AUC) to detect mortality. A plot of sensitivity versus 1-specificity was created using AUC in order to estimate the accuracy of these measurements. The optimal cut-off values were determined using ROC curves. The convergence point for the greatest sensitivity and specificity dictated the optimal cut-off points for each measurement. The AUC indicates the discriminative power of the test. Statistical significance was set at *p* < 0.05. The Kaplan–Meier product-limit estimator at 12 months was used to calculate the cumulative probability of death to estimate survival and to evaluate the difference among the PhA cut-off values. The Kaplan–Meier survival curves were compared using the log-rank (Mantel–Cox) test. The time of origin was the referred day. The event was defined as death, and all cases were censored at their last observation. 

In multivariate analysis, Cox proportional-hazards regression was used to assess the relationship between PhA and RFCSA and mortality in cancer patients. The hazard ratio (HR) and their 95% confidence intervals (CI) were calculated. The HR for death was expressed per 1° increase in PhA and 1 cm^2^ increase in RFCSA. To prevent potential confounding factors, the results were adjusted for several covariates that are known potential risk or protective factors for mortality: age (years, continuous); sex (man or woman); BMI (kg/m^2^, continuous); and CRP (mg/dl, continuous). We constructed an adjusted model with these variables. Statistical significance was set at *p* < 0.05.

## 3. Results

### 3.1. Baseline Characteristics

A total of 63 patients were referred from the Division of Oncology and began a comprehensive program for the morphofunctional assessment of DRM; 57 outpatients were included. Six patients were excluded for different reasons: not agreeing to participate (*n* = 4) and difficulty performing impedance (*n* = 2). The median (interquartile range) age was 62 years (54–70), predominantly men (61.4%). A total of 35% of patients had upper and lower gastrointestinal tract and hepatobiliary and pancreatic cancer, whereas 22.8% had lung cancer and 35% had other types (specified in Table 1). Of these, 36.8% received only chemotherapy, 26.2% other combination therapies, and 21.1% concomitant chemoradiotherapy. It should be noted that most of the patients (77.2%) were initially in stages III and IV. Nutritional assessment results were 71.9% at risk (Nutriscore), 68.4% with severe malnutrition (Score C, SGA), 8.8% malnourished (MNA), and with GLIM criteria, 94.2% had a diagnosis of malnutrition (Table 1). 

### 3.2. Morphofunctional Assessment Measurements between Survivor and Non-Survivor Patients at 12 Months

The morphofunctional assessment characteristics of the 12 months survivors and non-survivors are shown in Table 2. A comparative analysis was performed. There is a statistically significant difference in the anthropometric parameters and weight loss percentage between groups (11.9% in survivors vs. 14.9% in non-survivors; *p* = 0.041). The same difference was observed in quartiles of food intake assessment (75–100% food intake: 68.8% in survivors vs. 36%; *p* = 0.027). There was no difference between groups in age, weight, BMI, CC, and AMC (*p* > 0.05).

There is a statistically significant difference in the BIA between groups in PhA (5.4° in survivors vs. 4.7° in non-survivors; *p* < 0.001) and SphA (0.3 in survivors vs. −0.5 in non-survivors; *p* < 0.001). We also found differences in BCM (26.25 in survivors vs. 21.6 kg in non-survivors; *p* = 0.04). According to BIA parameters related to inflammation and hydration, there are differences in ECW/TBW (0.48 in survivors vs. 0.52 in non-survivors; *p* < 0.001) and TBW/FFM (73.4 in survivors vs. 73.7% in non-survivors; *p* = 0.033). There was no difference between groups in Rz/H, Xc/H, FFMI, FMI, ASMI, and ALM (*p* > 0.05).

**Figure 3:**
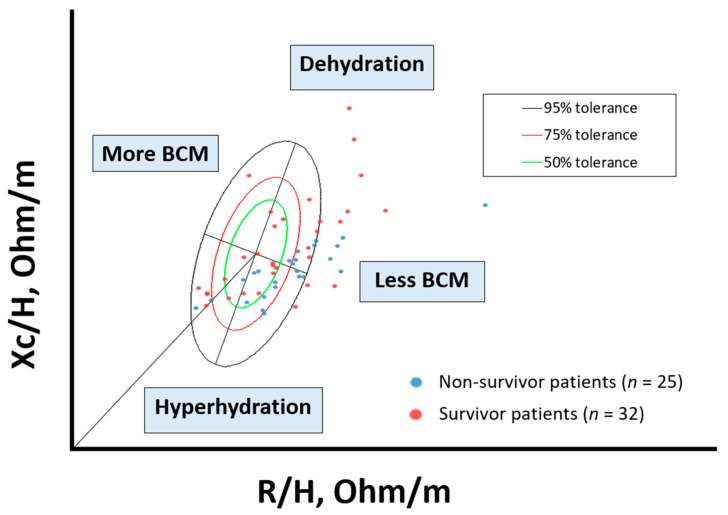
Scatterplot. Bioelectrical values of cancer disease: non-survivor patients (*n* = 25) and survivor patients (*n* = 32). BIA values: Rz/H (resistance/height, (Ohm/m)); Xc/H (reactance/height, (Ohm/h)), standardized by gender and age using bioelectrical Italian standards. The lower right quadrant encompasses patients with decreased body cell mass (BCM) and hyperhydration, most of the deceased patients. To interpret the color references in this figure legend, please see the Web version of this article. Source: own elaboration.

The distribution of individual impedance point vectors of the cancer patients shows a pattern of vector distribution in quadrants based on their nutritional and hydration characteristics (Figure 3). The vertical axis of the tolerance ellipse represents the degree of hydration, where an important part of patients are in a state of hyperhydration. The horizontal axis shows the cell mass; patients with low cell mass are associated with malnutrition. Non-survival patients are grouped in the lower right quadrant (hyperhydration and low BCM).

In relation to nutritional ultrasound®, there is a statistically significant difference in RFCSA between groups (4.27 in survivors vs. 2.98 cm^2^ in non-survivors; *p* = 0.03) and RF-Y-axis (1.3 in survivors vs. 0.9 cm in non-survivors; *p* = 0.007). There was no difference between groups in adipose tissue measures (*p* > 0.05). We did not find a difference between groups in HGS or functional tests, but we did in biochemical parameters in prealbumin (20.5 in survivors vs. 16.9 mg/dL in non-survivors; *p* = 0.018) and CRP/prealbumin (0.019 in survivors vs. 0.10 in non-survivors; *p* = 0.009). Finally, HRQoL showed statistically significant differences in EORTC-QLQ-C30 (58.33 in survivors vs. 66.66% in non-survivors; *p* = 0.007) but not in the NutriQoL®. 

### 3.3. Correlations between the Different Parameters of Morphofunctional Assessment of DRM

**Figure 4:**
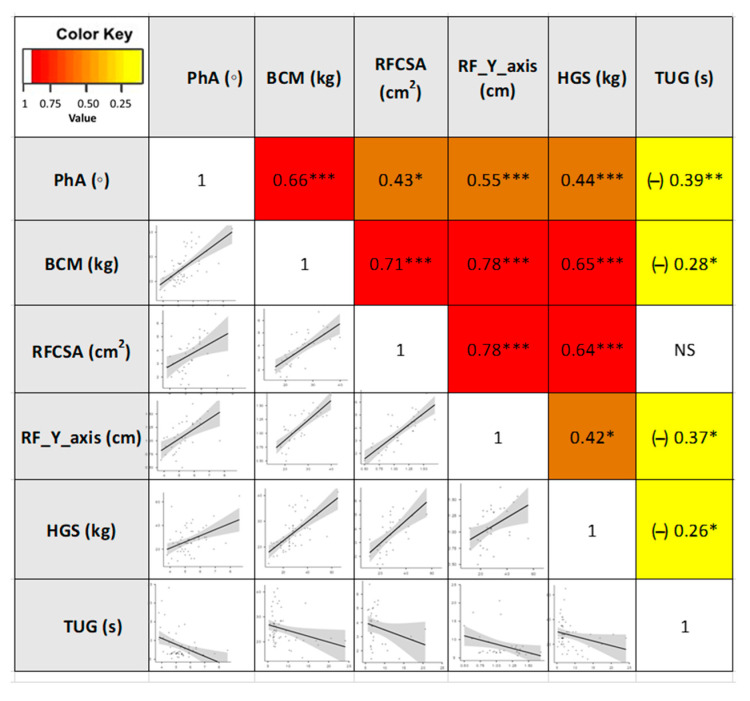
Heatmap correlation matrix and plot of the morphofunctional parameters in cancer patients. Abbreviations. PhA: phase angle; BCM: body cell mass; RFCSA: rectus femoris cross-sectional area; RF-Y-axis: rectus femoris Y axis; HGS: hand grip strength; TUG: Timed Up and Go test. Correlation describes the absolute value of r: 0.20–0.39 “weak”, r: 0.40–0.59 “moderate”, and 0.60–0.79 “strong”. * *p* < 0.05; ** *p* < 0.01; *** *p* < 0.001. NS: not significant To interpret the color references in this figure legend, please see the Web version of this article. Source: own elaboration.

These different approaches in the composition and function assessment based on different tools showed adequate correlations between the techniques (BIA, nutritional ultrasound®, HGS, and TUG). Correlation for PhA (BIA parameters) was observed with RFCSA (r = 0.43, *p* < 0.05) and RF-Y-axis (r = 0.55, *p* < 0.001) (nutritional ultrasound® parameters), and HGS (r = 0.44, *p* < 0.001). Other BIA parameters, such as BCM, showed a strong correlation with RFCSA (r = 0.71, *p* < 0.001) and RF-Y-axis (r = 0.78, *p* < 0.001). Using the Spearman correlation test, inverse correlations between morphological parameters and TUG were detected (Figure 4).

### 3.4. Optimal Morphofunctional Parameters of DRM Cut-Off Value and 12-Months Mortality

**Figure 5:**
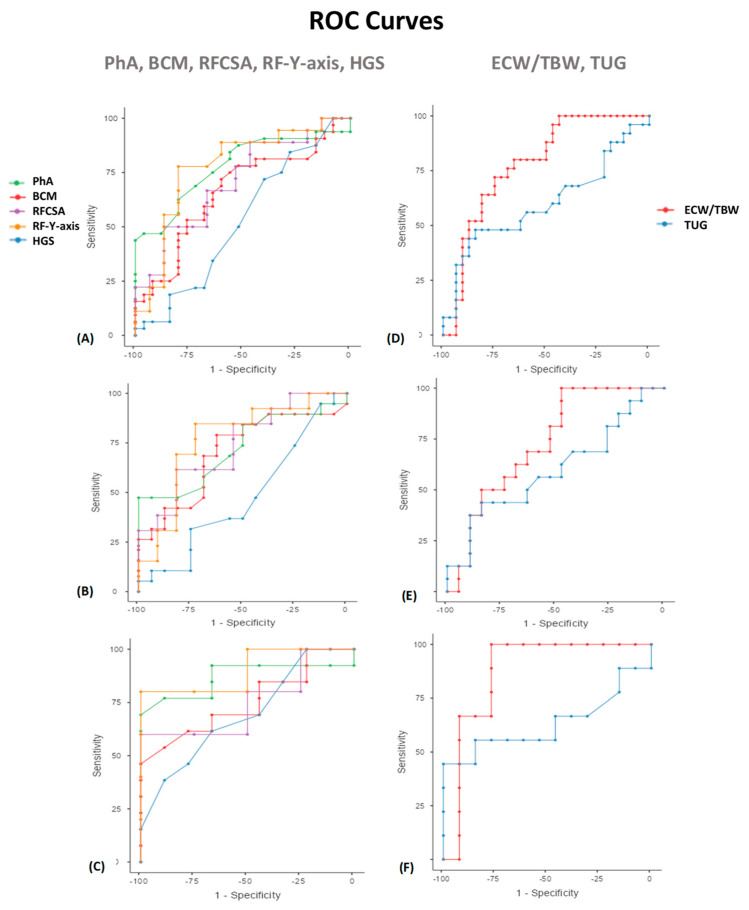
Area under the ROC curves (AUC) for predicting the survival at 12 months in cancer patients. PhA, BCM, RFCSA, RF-Y-axis, and HGS (**A**) Overall patients (**B**) Men (**C**) Women. ECW/TBW and TUG (**D**) Overall patients (**E**) Men (**F**) Women. ROC: receiver operating characteristic. Abbreviations. PhA: phase angle; BCM: body cell mass; RFCSA: rectus femoris cross-sectional area; RF-Y-axis: rectus femoris Y axis; HGS: hand grip strength; ECW/TBW: extracellular water/total body water; TUG: Timed Up and Go test. To interpret the color references in this figure legend, please see the Web version of this article. Source: own elaboration.

A ROC curve was constructed for analysis of the performance of PhA in predicting mortality (Table 3, Figure 5). In men, the AUC shows good discrimination (0.724; 95% CI, 0.50–0.95; *p* = 0.046). A PhA <5.9° shows a specificity of 100% and sensitivity of 47%. In women, the AUC shows good discrimination (0.868; 95% CI, 0.76–0.98; *p* = 0.05). A PhA <5.3° shows the highest specificity (100%) and sensitivity (69%) for mortality prediction. Evaluating the nutritional ultrasound® parameters, a ROC curve was constructed for analysis of the performance of RFCSA in predicting mortality. In men, the AUC shows good discrimination (0.741; 95% CI, 0.54–0.94; *p* = 0.046). An RFCSA <4.47° shows a specificity of 81.82% and sensitivity of 61.54%. In women, the AUC shows good discrimination (0.750; 95% CI, 0.53–0.97; *p* = 0.05). An RFCSA <2.73° shows the highest specificity (100%) and sensitivity (60%) for mortality prediction. We have also found useful cut-off values to predict 12-month mortality with other MFV parameters. The BIA parameters related to low cellular mass (BCM) and inflammation/hyperhydration (ECW/TBW) show a significant predictive value for mortality. This also occurs with the RF-Y-axis by nutritional ultrasound®. On the other hand, there are no clear cut-off values using ROC curves in the function parameters (HGS and TUG).

Evaluation of prognosis factors of mortality in cancer patients based on the area under the curve (AUC) of the receiver operating characteristic (ROC) curve and sensitivity and specificity values to determine the optimal cut-off values.

**Figure 6:**
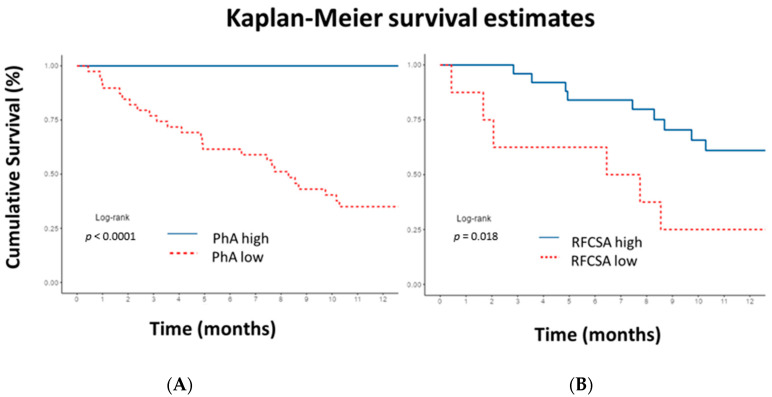
Kaplan–Meier 12-month survival plot illustrating cumulative survival for patients. (**A**) Low phase angle (PhA) in men <5.9° and in women <5.3°. (**B**) Low rectus femoris cross-sectional area (RFCSA) in men <4.47 cm^2^ and in women <2.73 cm^2^. Cut-off values obtained by receiver operating characteristic curve. *p*-values for log-rank test. A significantly lower survival rate was observed in patients with lower PhA and RFCSA cut-off values. To interpret the color references in this figure legend, please see the Web version of this article.

The Kaplan–Meier curve revealed that a PhA lower than 5.9° in men and 5.3° in women showed statistically significant (*p* < 0.0001) shorter survival time compared to higher PhA. Additionally, an RFCSA lower than 4.47 cm^2^ in men and 2.73 cm^2^ in women showed statistically significant (*p* = 0.018) shorter survival time compared to higher RFCSA (Figure 6). If the PhA is above the cut-off point, 12-month survival is 100%. If PhA is low, survival at 3, 6, and 12 months is 77% (65–91.4%, 95% CI), 62% (48–78.9%, 95% CI), and 35% (23–54.0%, 95% CI), respectively. On the other hand, if the RFCSA is above the cut-off point, survival at 3, 6, and 12 months is 96% (88.6–100%, 95% CI), 84% (70.8–99.7%, 95% CI), and 61% (43.9–84.9%, 95% CI). If the RFCSA is low, survival at 3, 6, and 12 months is 62.5% (36.5–100%, 95% CI), 62.5% (36.5–100%, 95% CI), and 25% (7.5–83%, 95% CI), respectively. The log-rank test revealed a difference between the curves (*p* < 0.001).

**Figure 7:**
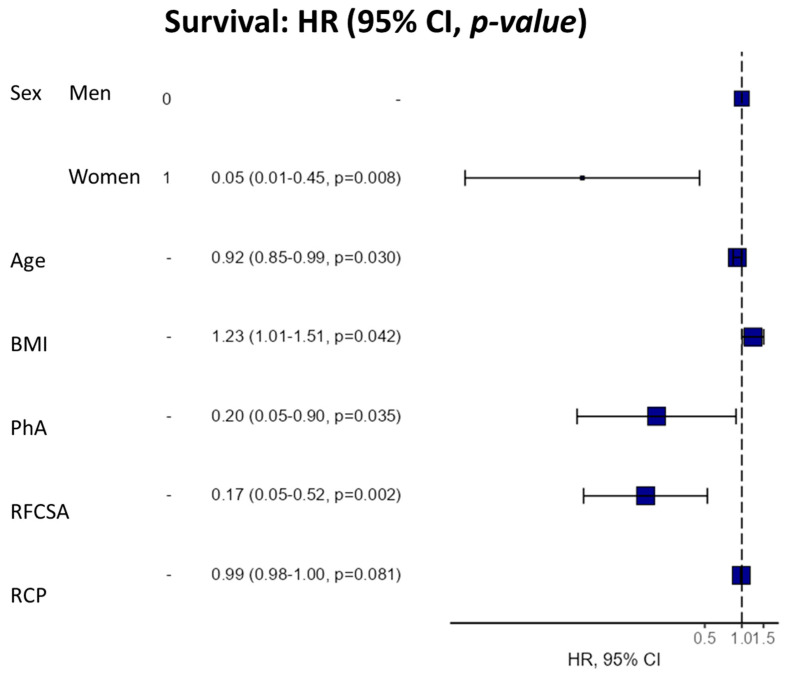
Hazard regression plot for survival (multivariable Cox analysis). Abbreviations. BMI: body mass index; PhA: phase angle; RFCSA: rectus femoris cross-sectional area; CRP: C-reactive protein.

We used a 6-component model multivariate analysis (by Cox regression) to evaluate the utility of the morphofunctional parameters as a prognostic factor for survival in cancer patients (Table 4). We found that high PhA was significantly associated with a lower mortality hazard ratio (HR 0.42 95% CI 0.21–0.84, *p* = 0.014). This trend was also maintained in the adjusted models by the confounding variables. Likewise, high RFCSA was associated with a decrease in mortality risk in the crude model (HR 0.61 95% CI 0.39–0.96, *p* = 0.031), with this relationship maintained in the adjusted models. Additionally, the multivariable survival analysis model revealed a strong relationship between PhA, RFCSA, BMI, and age, sex, and BMI (Figure 7).

The hazard ratio (HR) for survival was expressed per 1° increase in PhA and 1 cm^2^ increase in RFCSA for a univariate model and sequential adjustment models. Dependent variable: survivors (0) vs. non-survivors (1). Cox regression was expressed using HR and a 95% confidence interval (CI). NA (not applicable R-squared = 0.457 (Max possible = 0.945). Likelihood ratio test = 20.168 (df = 6, *p* = 0.003). Model 1: model adjusted for sex, age, BMI, and CRP. Abbreviations: BMI: body mass index; PhA: phase angle; RFCSA: rectus femoris cross-sectional area; CRP: C-reactive protein. 

## 4. Discussion

This prospective study showed that PhA, determined by BIA, and RFCSA, determined by ultrasound, are strong independent prognostic factors for mortality in a cohort of cancer patients. After adjusting for sex, age, BMI, and CRP, we determined that PhA and RFCSA were significant predictors of 12-month mortality in these patients. The use of the morphofunctional assessment of DRE allows the identification of patients at risk of mortality who are in particular need of intensified medical and nutritional care. Our results reinforce the correlations between different tools for nutritional assessment based on morphological (BIA and ultrasound) and functional (HGS and TUG) diagnosis.

BIA parameters have been widely described in the literature. PhA has an important prognostic factor for mortality in cancer patients in a heterogeneous population with different stages and without a clear stratification of the indicated therapeutic regimen. In our sample, patients are analyzed in an advanced state, mainly digestive location and at the beginning of antineoplastic treatment. In this sense, the diagnosis of nutritional risk is essential. The different nutritional tools highlight the usefulness of the recent GLIM criteria of DRM at 94.2%. GLIM has recently been launched [6], but it still needs validation studies in specific patient populations. Using the Investigation on Nutrition Status and Clinical Outcome of Common Cancers (INSCOC) cohort, Zhang et al. evaluated the efficacy of the GLIM to assess poor nutrition status, which is an independent risk factor for survival. The prevalence of malnutrition was 70.3%, which was similar to a previously published study [47]. Previous studies have shown the relationships between mortality and other indices (such as FFMI) [7]. In our study, we have not found any predictive cut-off value for mortality in relation to the anthropometric parameters obtained in BIA (Supplementary Material). Many studies have reported that PhA is a good indicator of mortality in cancer patients [48,49,50,51,52]. When analyzing BIA measurements in our cohort, higher PhA values were observed in cancer patients with a statistically significant difference between groups (5.4° in survivors vs. 4.7° in non-survivors; *p* < 0.001) (Table 2). Axelsson et al. reported similar results in a cohort of 128 patients (6.13° in survivors vs. 5.38° in non-survivors; *p* < 0.001).

Beyond the absolute value of PhA, our work highlights the importance of evaluating the following vector positions in the RXc graph. Longitudinal changes in hydration and cell mass are, therefore, interpreted more reliably by BIVA than PhA alone [53].

BIVA can indirectly estimate body composition, representing a useful and non-invasive nutrition assessment in cancer patients. Mueller et al., in a large cohort, were able to demonstrate that BIA values correlate significantly with values from CT analysis independent of the patient’s nutritional status [54]. In a single-center study, Katsura et al. showed that the ECW/TBW ratio was associated with mortality (HR per 1-IQR increase: 2.87; 95% CI: 1.46, 5.46; *p* < 0.001) in patients with cancer cachexia [55].

Among the emerging parameters of morphofunctional assessment, the use of ultrasound to assess muscle mass and adipose tissue is emphasized. We found differences in RFCSA and RF-Y-axis between survival and non-survivor patients. Higher RFCSA values were observed in cancer patients with a statistically significant difference between groups (4.27 in survivors vs. 2.98 cm^2^ in non-survivors; *p* = 0.03) (Table 2). Galli et al. showed findings in the same direction, with low RFCSA being an independent prognostic factor for lower overall survival both at a multivariate analysis (OR 4.42, 95% CI 1.12–17.40; *p* = 0.033), even after adjustment for different factors [56]. Bril et al. showed that by using other imaging techniques, such as CT and MRI scans, in their 235-case cohort of total laryngectomy patients, a low skeletal muscle mass (SMM) was predictive of poor prognosis at multivariate Cox regression analysis (OR 2.096, 95% CI 1.494–2.920; *p* < 0.001) [57]. Different studies relate the risk of mortality in cancer patients with functional tests. The HGS has more evidence, but we have not found significant differences, which could be related to the size of the sample.

Moreover, significant correlations were found between different tools for nutritional assessment. Our study highlights a strong correlation between emerging techniques of ultrasound (RFCSA and RF-Y-axis) with the more established parameters of BIA (PhA and BCM). Formenti et al. recently showed a lack of correlation between BIVA variables and muscle ultrasound in critically ill patients. It should be considered that during the acute phase of a severe catabolic illness, normal muscle is gradually replaced by fat or fibrous tissues [58]. In our patients with low-grade chronic inflammation associated with the tumor, the assessment of muscle size may be more reliable than in patients with severe inflammatory conditions. We also found a moderate correlation between PhA and HGS (r = 0.44), as described in the study by Souza et al. (r = 0.54) in a series of cancer patients with a similar age and tumor stage to our series.

PhA was a predictor of muscle abnormalities and function and had good diagnostic accuracy for detecting low muscle mass using computerized tomography imaging [59].

In our study, we found that PhA (OR = 6.11, *p* = 0.042) and RFCSA (OR = 6.35, *p* = 0.032) were significant predicting factors for mortality in cancer patients. The PhA cut-off point values obtained with ROC analysis in our sample were <5.3° in women and <5.9° in men, which differs from other cohorts that proposed a cut-off point without considering differences between sexes. Garlini et al., in their systematic review, presented less variation in the cut-off point, <4.4° to <5.8° in cancer patients [48]. Galli et al. defined a low RFCSA < 0.97 cm^2^ adjusted by sex (AUC 0.751, 95% CI 0.607–0.895) based on 30-day postoperative complications. In our study, the cut-off value is higher and more accurate due to the specific value of each sex (2.73 cm^2^ in women and 4.47 cm^2^ in men). Da Silva et al. showed that RFCSA (≤5.9 cm^2^/m^2^) was the only independent predictor of 28-day mortality (OR = 6.08; *p* = 0.028) [60].

The Kaplan–Meier estimate for overall survival according to the sex-adjusted RFCSA cut-off showed a low survival rate at 12 months in our cancer patients. Similar findings are shown in other studies [56]. Additionally, Axelsson et al. found that patients with the lower half of median PhA values (5.58°) had a lower significant prognostic factor for mortality (HR 0.47, *p* < 0.001) [49].

In the mortality analysis, it is important to highlight the profile of deceased patients. It shows a significant decrease in PhA and RFCSA, indicating that a deteriorating nutritional status is associated with a risk of mortality after a multivariable regression analysis. 

Bioelectric and ultrasound results obtained in cancer patients who had poor prognosis and eventually died give us information about the presence of cell damage (decrease in PhA) and nutritional impairment (low RFCSA), which may be useful as a prognostic factor for mortality and direct therapeutic interventions in a more individualized way.

The strength of this study is that it is the first time that different morphofunctional assessment tools have been integrated, and clinicians can make more accurate diagnoses and therapies. 

Morphofunctional parameters provide individual prognostic information for each patient, regardless of established clinical parameters, such as tumor staging and ECOG. Therefore, they can show the ability to individualize prognoses and treatment plans. In addition, they are factors linked to the nutritional situation, depending on the greater or lesser conservation of lean mass and potentially reversible with nutritional intervention and functional physical recovery. Groups of patients with severe morphofunctional compromise may be candidates for a more aggressive approach to improve their prognosis. If patients show low values of PhA, BCM, RFCSA, and HGS, personalizing the therapeutic approach for each patient will be assessed.

We have several limitations in our study. The first is the heterogeneity of the population under analysis regarding tumor entity and type of treatment, which weakens the results. The second is the small sample size of patients analyzed. Third, no consensus exists regarding cut-off values for PhA and RFCSA, limiting the possibility of systematic comparison with previous results. Other limitations are that if these techniques are going to be adopted for clinical use, the methods must be available to the oncologist. In addition, it is necessary to establish cut-off values for PhA and RFCSA for different patients. However, our study highlights that PhA and RFCSA are important independent predictors of mortality in cancer patients. Nevertheless, considering the observational nature of the study design, this does not imply a causality relationship, and on the other hand, data were exposed to possible residual unmeasured confounders. 

## 5. Conclusions

PhA and RFCSA seem to be promising and useful independent predictors of mortality in cancer patients. Low values of these parameters were significantly associated with shorter overall survival. In our study, we found that PhA (OR = 6.11, *p* = 0.042) and RFCSA (OR = 6.35, *p* = 0.032) were significant predicting factors for mortality in cancer patients. The PhA cut-off point obtained using ROC analysis in our sample was <5.3° in women and <5.9° in men. Additionally, RFCSA lower than 4.47 cm^2^ in men and 2.73 cm^2^ in women showed shorter survival time. Cut-off points are clinical data that can be used for nutritional assessment and the prediction of clinical outcomes. Higher PhA values were observed in cancer patients with a statistically significant difference between groups (5.4° in survivors vs. 4.7° in non-survivors; *p* < 0.001). Higher RFCSA values were observed in cancer patients with a statistically significant difference between groups (4.27 in survivors vs. 2.98 cm^2^ in non-survivors; *p* = 0.03).

This study conceptualizes the morphofunctional assessment of DRM, that is, the morphological aspects of the size of the cell mass and function. The novelty or added value is that it is a global vision that can help classify patients who require multimodal interventions to improve their results. The present findings support the use of this assessment in clinical practice to help clinicians identify patients at risk of malnutrition. It allows assessing in-depth body composition and function to provide more nutritional attention, improve their HRQoL, and generate real-world evidence in future research lines. 

## Figures and Tables

**Table 1:** Baseline clinical characteristics.

Variables	Baseline
**Primary site tumor** Lung Hepatobiliary and Pancreatic Upper gastrointestinal tract Lower gastrointestinal tract Other: Urologic Sarcoma Breast cancer Gynecologic cancer Hematologic cancer Oral cancer	**Median (IQR)** 62 (54–70)
**Treatment** Only chemotherapy Only radiotherapy Only surgery Biological therapy Concomitant chemoradiotherapy Other combination therapies	***n* (%)** 35 (61.4) 22 (38.6)
**Tumor stage** I II III IV	
**ECOG** 0 1 2	***n* (%)** 13 (22.8) 10 (17.5) 7 (12.3) 7 (12.3) 20 (35.1) 5 (8.8) 5 (8.8) 4 (7) 3 (5.3) 2 (3.5) 1 (1.7)
**Primary site tumor** Lung Hepatobiliary and Pancreatic Upper gastrointestinal tract Lower gastrointestinal tract Other: Urologic Sarcoma Breast cancer Gynecologic cancer Hematologic cancer Oral cancer	***n* (%)** 21 (36.8) 1 (1.8) 3 (5.3) 5 (8.8) 12 (21.1) 15 (26.2)
**Treatment** Only chemotherapy Only radiotherapy Only surgery Biological therapy Concomitant chemoradiotherapy Other combination therapies	***n* (%)** 2 (3.5) 11 (19.3) 14 (24.6) 30 (52.6)
**Tumor stage** I II III IV	***n* (%)** 25 (43.9) 24 (42.1) 8 (14)
**Nutritional assessment**	
**Nutriscore** Without risk At risk	***n* (%)** 16 (28.1) 41 (71.9)
**Subjective Global Assessment (SGA)** Normally nourished (A) Moderate malnutrition (B) Severe malnutrition (C)	***n* (%)** 11 (19.3) 7 (12.3) 39 (68.4)
**Mini Nutritional Assessment (MNA)** Normal nutritional status At risk of malnutrition Malnourished	***n* (%)** 23 (40.4) 29 (50.9) 5 (8.8)
**GLIM criteria** **Phenotypic criteria** Weight loss (kg; >5% within past 6 months) Low BMI (kg/m^2^; <20 if <70 years or <22 if >70 years) Reduced muscle mass: By BIA Low FFMI (kg/m^2^; <17 males/15 females) Low ASMI (kg/m^2^; <7 males/<5.7 females) Low ALM (kg; <21.4 males/<14.1 females) By anthropometry CC (cm; <34 males/<33 females) AMC (cm; <p5) **Etiologic criteria** Reduced food intake (≤50% for energetic requirements >1 week) Disease burden/inflammation **Diagnosis of malnutrition (1 phenotypic + 1 etiologic criteria)**	***n* (%)** 47 (82.5) 20 (35.1) 10 (17.5) 0 28 (49.1) 49 (86.0) 10 (17.5) 12 (21.1) 57 (100) 56 (94.2)

Abbreviations: IQR: interquartile range; BMI: body mass index; BIA: bioelectrical impedance analysis; FFMI: fat-free mass index; ASMI: appendicular skeletal muscle index; ALM: appendicular lean mass; CC: calf circumference; AMC: arm muscle circumference.

**Table 2:** Morphofunctional parameters of cancer patients related to survival and mortality.

		Cancer Patients	Cancer Patient Survivors at 1 Year	Cancer Patient Non-Survivors at 1 Year	^1^ *p*
		Median (IQR)	Median (IQR)	Median (IQR)	(* *p* < 0.05; ** *p* < 0.01; *** *p* < 0.001)
		*n* = 57	*n* = 32	*n* = 25	
Anthropometric parameters					
	Age	62 (54–70)	62 (52.7–72)	62 (57–70)	0.901
	Weight (kg)	60.3 (54.1–73.1)	60.6 (54.5–74.5)	60.3 (50.6–72.2)	0.469
	Height (cm)	169 (160–175)	169 (160–175)	168 (160–173)	0.449
	BMI (kg/m^2^)	23 (19.9–25.5)	22.6 (20–25.7)	23 (20–25.4)	0.537
	Weight loss (%)	12.5 (6.5–17.9)	11.9 (4.9–14.7)	14.9 (7.4–22)	0.041 *
	CC (cm)	31 (30–34)	31.5 (30–35)	30 (28–32)	0.232
	AMC (cm)	22.2 (20.2–23.2)	22.2 (20.7–23.9)	20.7 (20–23.2)	0.442
Food intake assessment					
	0–25%	6 (10.5%)	1 (3.1%)	5 (20%)	0.027 *
	25–50%	6 (10.5%)	4 (12.5%)	2 (8%)	
	50–75%	14 (24.6%)	5 (15.6%)	9 (36%)	
	75–100%	31 (54.4%)	22 (68.8%)	9 (36%)	
Bioelectrical Impedance Analysis (BIA)					
	PhA (◦)	5.2 (4.7–5.6)	5.4 (5.07–6.2)	4.7 (4.5–5.2)	<0.001 ***
	SPhA	0.1 (−0.8–0.9)	0,3 (−0.2–1.3)	−0,5 (−1.5–0.3)	<0.001 ***
	Rz/H (Ω/m)	319.4 (290.1–356.8)	310.6 (279–358.8)	330 (296–355)	0.803
	Xc/H (Ω/m)	28.4 (25–32.8)	29.7 (25.1–36)	27.5 (25–29.4)	0.066
	BCM (kg)	23.9 (20.7–28.2)	26.25 (22.05–29.35)	21.6 (20.5–24.7)	0.040 *
	BCM/H (kg/m)	14.1 (12.9–16.8)	15.3 (13.7–17.5)	13,3 (12.5–14.8)	0.015 *
	FFMI (kg/m^2^)	17.6 (16.3–19.1)	17.9 (16.4–19.2)	17.5 (15.9–18.9)	0.590
	FMI (kg/m^2^)	4.5 (3–6.1)	4.6 (2.9–6.1)	4.4 (3–6.2)	0.847
	ASMI (kg/m^2^)	8.6 (7.7–9.6)	8.5 (7.3–9.7)	8.7 (7.8–9.4)	0.886
	ALM (kg)	18.3 (15.7–21.8)	18.65 (15.92–22.07)	18 (15.2–20.3)	0.347
	ECW/TBW	0.5 (0.47–0.53)	0.48 (0.44–0.50)	0.52 (0.5–0.54)	<0.001 ***
	TBW/FFM (%)	73.6 (73.2–73.8)	73.4 (73.07–73.7)	73.7 (73.5–73.9)	0.033 *
Nutritional ultrasound®: rectus femoris muscle					
	RFCSA (cm^2^)	3.7 (2.5–4.6)	4,27 (3.06–4.96)	2.98 (2.30–4.12)	0.030 *
	RF- Circumference (cm)	8.8 (7.9–10)	9.69 (8.08–10.18)	8.61 (7.59–9.47)	0.186
	RF-X-axis (cm)	3.7 (3.5–4.3)	3.98 (3.42–4.34)	3,66 (3.48–4.1)	0.369
	RF-Y-axis (cm)	1.1 ( 0.9–1.3)	1,30 (1.06–1.34)	0.9 (0.74–1.05)	0.007 **
	RF-Adipose tissue (cm)	0.5 (0.3–0.7)	0.52 (0.35–0.74)	0.5 (0.32–0.67)	0.553
Nutritional ultrasound®: abdominal adipose tissue					
	Superficial subcutaneous (cm)	0.63 (0.38–0.82)	0.68 (0.51–0.98)	0.59 (0.29–0.66)	0.349
	Total subcutaneous (cm)	1.31 (0.84–1.72)	1.33 (0.84–1.72)	1.17 (0.59–1.66)	0.724
	Preperitoneal visceral (cm)	0.55 (0.38–0.72)	0.55 (0.36–0.61)	0.54 (0.38–0.73)	1.000
Hand Grip Strength					
	Hand Grip Strength (kg)	25 (20–34)	26.5 (21.5–30)	25 (18–35)	0.746
Functional tests					
	Timed Up and Go Test (s)	6.83 (6.5–8.7)	6.82 (6.5–7.8)	7.1 (6.4–11.9)	0.192
Biochemical parameters					
	Prealbumin (mg/dL)	20 (14.9–25.9)	20.5 (15.92–28.35)	16.9 (10.2–22.7)	0.018 *
	CRP/Prealbumin	0.04 (0.01–0.1)	0.019 (0.012–0.053)	0.10 (0.03–0.17)	0.009 **
HRQoL					
	EORTC QLQ C30 (global) (%)	58.3 (41.7–83.3)	58.33 (41.66–83.33)	66.66 (50–75)	0.009 **
	NutriQoL® (total score) (%)	88.2 (79.4–97)	91.18 (74.5–97.06)	84.8 (79.41–93.38)	0.619

Abbreviations. ^1^ *p* for comparison of non-survivors and survivors. IQR: interquartile range; BMI: body mass index; CC: calf circumference; AMC: arm muscle circumference; PhA: phase angle; SPhA: standardized phase angle; Rz/H: resistance/height; Xc/H: reactance/height; BCM: body cell mass; BCM/H: body cell mass/height; FFMI: fat-free mass index; FMI: fat mass index; appendicular skeletal muscle index: ASMI; appendicular lean mass: ALM; ECW/TBW: extracellular water/total body water; TBW/FFM: total body water/fat-free mass; RFCSA: rectus femoris cross-sectional area; CRP/prealbumin: C-reactive protein/prealbumin.

**Table 3:** ROC curve analysis of the prognostic factor of mortality in cancer patients.

Variables		Cut Off Point	AUC	Sensitivity	Specificity	PPV (%)	NPV (%)	*p*
PhA (◦)	PhA (◦)	5.6	0.803	55.56	96.67	93.75	70.73	0.009 **
	PhA Men	5.9	0.724	47.37	100	100	61.54	0.046 *
	PhA Women	5.3	0.868	69.23	100	100	69.23	0.05 *
BCM (kg)	BCM (kg)	22.3	0.661	71.88	60	69.7	62.5	NS
	BCM Men	26.2	0.702	78.95	62.5	71.43	71.43	0.05 *
	BCM Women	22.3	0.752	46.15	100	100	56.25	0.05 *
ECW/TBW	ECW/TBW	0.5	0.780	72	75	69.23	77.42	0.041 *
	ECW/TBW Men	0.47	0.730	100	47.37	61.54	100	0.048 *
	ECW/TBW Women	0.5	0.872	100	76.92	75	100	0.045 *
RFCSA (cm^2^)	RFCSA (cm^2^)	4.47	0.722	60	88.89	81.82	72.73	0.043 *
	RFCSA Men	4.47	0.741	61.54	81.82	80	64.29	0.046 *
	RFCSA Women	2.73	0.750	60	100	100	66.67	0.05 *
RF-Y-axis (cm)	RF-Y-axis (cm)	1.3	0.735	60	83.33	75	71.43	0.007 *
	RF-Y-axis Men	1.06	0.769	84.62	72.73	78.57	80	0.026 *
	RF-Y-axis Women	1	0.800	80	100	100	80	0.05 *
HGS (kg)	HGS (kg)	20	0.581	88.89	30	53.33	75	NS
	HGS Men	25	0.477	94.74	12.5	56.25	66.67	NS
	HGS Women	20	0.700	61.54	66.67	72.73	54.55	0.05 *
TUG (s)	TUG (s)	8.2	0.602	48	84.38	70.59	67.5	NS
	TUG Men	8.2	0.599	43.75	84.21	70	64	NS
	TUG Women	10.76	0.632	44.44	100	100	72.22	0.05 *

Abbreviations: AUC: area under the curve; PPV: positive predictive value; NPV: negative predictive value; PhA: phase angle; BCM: body cell mass; ECW/TBW: extracellular water/total body water; RFCSA: rectus femoris cross-sectional area; RF-Y-axis: rectus femoris Y axis; HGS: hand grip strength; TUG: Timed Up and Go test. * *p* < 0.05; ** *p* < 0.01.

**Table 4:** Multivariable analysis of the prognostic factor for mortality in cancer patients.

Dependent	HR (Univariable)	HR (Multivariable)
Sex (Male-Female)	0.91 (0.29–2.86, *p* = 0.870)	0.05 (0.01-0.45, *p* = 0.008)
Age	1.00 (0.96–1.04, *p* = 0.983)	0.92 (0.85–0.99, *p* = 0.030)
BMI	0.96 (0.85–1.08, *p* = 0.459)	1.23 (1.01–1.51, *p* = 0.042)
PhA	0.42 (0.21–0.84, *p* = 0.014)	0.20 (0.05–0.90, *p* = 0.035)
RFCSA	0.61 (0.39–0.96, *p* = 0.031)	0.17 (0.05–0.52, *p* = 0.002)
CRP	1.00 (1.00–1.01, *p* = 0.169)	0.99 (0.98–1.00, *p* = 0.081)

## Data Availability

The data that support the findings of this study are available from the corresponding author upon reasonable request.

## Supplementary Materials

The following supporting information can be downloaded at: https://www.mdpi.com/article/10.3390/nu15030522/s1, Figure S1: ROC Curve.; Table S1: Anthropometric parameters obtained in BIA.

## References

[1] Muscaritoli M., Arends J., Bachmann P., Baracos V., Barthelemy N., Bertz H., Bozzetti F., Hütterer E., Isenring E., Kaasa S. (2021). ESPEN practical guideline: Clinical Nutrition in cancer. Clin. Nutr. Edinb. Scotl..

[2] Arends J., Baracos V., Bertz H., Bozzetti F., Calder P.C., Deutz N.E.P., Erickson N., Laviano A., Lisanti M.P., Lobo D.N. (2017). ESPEN expert group recommendations for action against cancer-related malnutrition. Clin. Nutr. Edinb. Scotl..

[3] Fearon K., Strasser F., Anker S.D., Bosaeus I., Bruera E., Fainsinger R.L., Jatoi A., Loprinzi C., MacDonald N., Mantovani G. (2011). Definition and classification of cancer cachexia: An international consensus. Lancet Oncol..

[4] Cruz-Jentoft A.J., Bahat G., Bauer J., Boirie Y., Bruyère O., Cederholm T., Cooper C., Landi F., Rolland Y., Sayer A.A. (2019). Sarcopenia: Revised European consensus on definition and diagnosis. Age Ageing.

[5] Meza-Valderrama D., Marco E., Dávalos-Yerovi V., Muns M.D., Tejero-Sánchez M., Duarte E., Sánchez-Rodríguez D. (2021). Sarcopenia, Malnutrition, and Cachexia: Adapting Definitions and Terminology of Nutritional Disorders in Older People with Cancer. Nutrients.

[6] Cederholm T., Jensen G.L., Correia M.I.T.D., Gonzalez M.C., Fukushima R., Higashiguchi T., Baptista G., Barazzoni R., Blaauw R., Coats A. (2019). GLIM criteria for the diagnosis of malnutrition–A consensus report from the global clinical nutrition community. Clin. Nutr..

[7] Zhang X., Li X., Shi H., Zhang K., Zhang Q., Tang M., Li W., Zhou F., Liu M., Cong M. (2022). Association of the fat-free mass index with mortality in patients with cancer: A multicenter observational study. Nutr. Burbank Los. Angel Cty. C. Calif..

[8] García Almeida J.M., García García C., Vegas Aguilar I.M., Bellido Castañeda V., Bellido Guerrero D. (2021). Morphofunctional assessment of patient´s nutritional status: A global approach. Nutr. Hosp..

[9] Prado C.M., Sawyer M.B., Ghosh S., Lieffers J.R., Esfandiari N., Antoun S., Baracos V.E. (2013). Central tenet of cancer cachexia therapy: Do patients with advanced cancer have exploitable anabolic potential?. Am. J. Clin. Nutr..

[10] Thomas D.R. (2007). Loss of skeletal muscle mass in aging: Examining the relationship of starvation, sarcopenia and cachexia. Clin. Nutr. Edinb. Scotl..

[11] Surov A., Pech M., Gessner D., Mikusko M., Fischer T., Alter M., Wienke A. (2021). Low skeletal muscle mass is a predictor of treatment related toxicity in oncologic patients. A meta-analysis. Clin. Nutr. Edinb. Scotl..

[12] de Oliveira Faria S., Alvim Moravia R., Howell D., Eluf Neto J. (2021). Adherence to nutritional interventions in head and neck cancer patients: A systematic scoping review of the literature. J. Hum. Nutr. Diet..

[13] Ravasco P. (2019). Nutrition in Cancer Patients. J. Clin. Med..

[14] Gupta D., Lammersfeld C.A., Burrows J.L., Dahlk S.L., Vashi P.G., Grutsch J.F., Hoffman S., Lis C.G. (2004). Bioelectrical impedance phase angle in clinical practice: Implications for prognosis in advanced colorectal cancer. Am. J. Clin. Nutr..

[15] Arribas L., Hurtós L., Sendrós M.J., Peiró I., Salleras N., Fort E., Sánchez-Migallón J.M. (2017). NUTRISCORE: A new nutritional screening tool for oncological outpatients. Nutr. Burbank. Los Angel Cty Calif..

[16] Detsky A.S., McLaughlin J.R., Baker J.P., Johnston N., Whittaker S., Mendelson R.A., Jeejeebhoy K.N. (1987). What is subjective global assessment of nutritional status?. JPEN J. Parenter. Enteral. Nutr..

[17] Vellas B., Villars H., Abellan G., Soto M.E., Rolland Y., Guigoz Y. (2006). Overview Of The Mna®–Its History And Challenges. J. Nutr. Health Aging.

[18] Cederholm T., Bosaeus I., Barazzoni R., Bauer J., Van Gossum A., Klek S., Muscaritoli M., Nyulasi I., Ockenga J., Schneider S.M. (2015). Diagnostic criteria for malnutrition—An ESPEN Consensus Statement. Clin. Nutr. Edinb. Scotl..

[19] Alaustré A., Rull M., Camps I., Ginesta C., Melus M., Salvá J. (1988). Nuevas normas y consejos en la valoración de los parámetros en nuestra población: Índice adiposo-muscular, índice ponderales y tablas de percentiles de los datos antropométricos útiles en una valoración nutricional. Med. Clin..

[20] Gonzalez M.C., Mehrnezhad A., Razaviarab N., Barbosa-Silva T.G., Heymsfield S.B. (2021). Calf circumference: Cutoff values from the NHANES 1999-2006. Am. J. Clin. Nutr..

[21] Fernández García-Salazar R., García-Almeida J.M. (2016). Valoración del estado nutricional y concepto de desnutrición. Olveira G. Manual de Nutrición Clínica Y Dietética.

[22] Villalobos Gámez J., García-Almeida J., del Río Mata J., Rioja Vázquez R., Barranco Pérez J., Bernal Losada O., García-Almeida J.M., Villalobos Gámez J. (2012). Proceso INFORNUT®. Minivademecum Nutricional.

[23] Lukaski H.C., Bolonchuk W.W., Hall C.B., Siders W.A. (1986). Validation of tetrapolar bioelectrical impedance method to assess human body composition. J. Appl. Physiol..

[24] Dunbar C.C., Melahrinides E., Michielli D.W., Kalinski M.I. (1994). Effects of small errors in electrode placement on body composition assessment by bioelectrical impedance. Res. Q. Exerc. Sport.

[25] Dixon C.B., LoVallo S.J., Andreacci J.L., Goss F.L. (2006). The effect of acute fluid consumption on measures of impedance and percent body fat using leg-to-leg bioelectrical impedance analysis. Eur. J. Clin. Nutr..

[26] Piccoli A., Rossi B., Pillon L., Bucciante G. (1994). A new method for monitoring body fluid variation by bioimpedance analysis: The RXc graph. Kidney. Int..

[27] Piccoli A., Nigrelli S., Caberlotto A., Bottazzo S., Rossi B., Pillon L., Maggiore Q. (1995). Bivariate normal values of the bioelectrical impedance vector in adult and elderly populations. Am. J. Clin. Nutr..

[28] De Palo T., Messina G., Edefonti A., Perfumo F., Pisanello L., Peruzzi L., Di Iorio B., Mignozzi M., Vienna A., Conti G. (2000). Normal values of the bioelectrical impedance vector in childhood and puberty. Nutr. Burbank. Los Angel Cty Calif..

[29] Paiva S.I., Borges L.R., Halpern-Silveira D., Assunção M.C.F., Barros A.J.D., Gonzalez M.C. (2010). Standardized phase angle from bioelectrical impedance analysis as prognostic factor for survival in patients with cancer. Support. Care Cancer.

[30] Schutz Y., Kyle U.U.G., Pichard C. (2002). Fat-free mass index and fat mass index percentiles in Caucasians aged 18-98 y. Int. J. Obes..

[31] Moore F.D., Boyden C.M. (1963). Body Cell Mass And Limits Of Hydration Of The Fat-Free Body: Their Relation To Estimated Skeletal Weight. Ann. NY Acad. Sci. USA.

[32] Hernández-Socorro C.R., Saavedra P., López-Fernández J.C., Ruiz-Santana S. (2018). Assessment of Muscle Wasting in Long-Stay ICU Patients Using a New Ultrasound Protocol. Nutrients.

[33] Perkisas S., Bastijns S., Sanchez-Rodriguez D., Piotrowicz K., De Cock A.-M. (2021). Application of ultrasound for muscle assessment in sarcopenia: 2020 SARCUS update: Reply to the letter to the editor: SARCUS working group on behalf of the Sarcopenia Special Interest Group of the European Geriatric Medicine Society. Eur. Geriatr. Med..

[34] Hamagawa K., Matsumura Y., Kubo T., Hayato K., Okawa M., Tanioka K., Yamasaki N., Kitaoka H., Yabe T., Nishinaga M. (2010). Abdominal visceral fat thickness measured by ultrasonography predicts the presence and severity of coronary artery disease. Ultrasound. Med. Biol..

[35] Pérez Miguelsanz M.J., Cabrera Parra W., Varela Moreiras G., Garaulet M. (2010). Regional distribution of the body fat: Use of image techniques as tools for nutritional diagnosis. Nutr. Hosp..

[36] Sanz-Paris A., González-Fernandez M., Hueso-Del Río L.E., Ferrer-Lahuerta E., Monge-Vazquez A., Losfablos-Callau F., Sanclemente-Hernández T., Sanz-Arque A., Arbones-Mainar J.M. (2021). Muscle Thickness and Echogenicity Measured by Ultrasound Could Detect Local Sarcopenia and Malnutrition in Older Patients Hospitalized for Hip Fracture. Nutrients.

[37] Sánchez Torralvo F.J., Porras N., Abuín Fernández J., García Torres F., Tapia M.J., Lima F., Soriguer F., Gonzalo M., Rojo Martínez G., Olveira G. (2018). Normative reference values for hand grip dynamometry in Spain. Association with lean mass. Nutr. Hosp..

[38] Podsiadlo D., Richardson S. (1991). The timed ‘Up & Go’: A test of basic functional mobility for frail elderly persons. J. Am. Geriatr. Soc..

[39] Steffen T.M., Hacker T.A., Mollinger L. (2002). Age- and gender-related test performance in community-dwelling elderly people: Six-Minute Walk Test, Berg Balance Scale, Timed Up & Go Test, and gait speeds. Phys. Ther..

[40] Beck F.K., Rosenthal T.C. (2002). Prealbumin: A marker for nutritional evaluation. Am. Fam. Physician.

[41] Pinilla J.C., Hayes P., Laverty W., Arnold C., Laxdal V. (1998). The C-reactive protein to prealbumin ratio correlates with the severity of multiple organ dysfunction. Surgery.

[42] Kim M.-R., Kim A.-S., Choi H.-I., Jung J.-H., Park J.Y., Ko H.-J. (2020). Inflammatory markers for predicting overall survival in gastric cancer patients: A systematic review and meta-analysis. PloS ONE.

[43] Li L., Dai L., Wang X., Wang Y., Zhou L., Chen M., Wang H. (2017). Predictive value of the C-reactive protein-to-prealbumin ratio in medical ICU patients. Biomark Med..

[44] Aaronson N.K., Ahmedzai S., Bergman B., Bullinger M., Cull A., Duez N.J., Filiberti A., Flechtner H., Fleishman S.B., de Haes J.C. (1993). The European Organization for Research and Treatment of Cancer QLQ-C30, a quality-of-life instrument for use in international clinical trials in oncology. J. Natl. Cancer Inst..

[45] Apezetxea A., Carrillo L., Casanueva F., Cuerda C., Cuesta F., Irles J., Virgili M., Virgili M., Lizán L. (2016). The NutriQoL® questionnaire for assessing health-related quality of life (HRQoL) in patients with home enteral nutrition (HEN): Validation and first results. Nutr. Hosp..

[46] Wanden-Berghe C., Cheikh Moussa K., Sanz-Valero J. (2018). Adherencia a la Nutrición Enteral Domiciliaria. Hosp. Domic..

[47] Zhang Q., Zhang K.-P., Zhang X., Tang M., Song C.-H., Cong M.-H., Guo Z.-Q., Ding J.-S., Braga M., Cederholm T. (2021). Scored-GLIM as an effective tool to assess nutrition status and predict survival in patients with cancer. Clin. Nutr. Edinb. Scotl..

[48] Garlini L.M., Alves F.D., Ceretta L.B., Perry I.S., Souza G.C., Clausell N.O. (2019). Phase angle and mortality: A systematic review. Eur. J. Clin. Nutr..

[49] Axelsson L., Silander E., Bosaeus I., Hammerlid E. (2018). Bioelectrical phase angle at diagnosis as a prognostic factor for survival in advanced head and neck cancer. Eur Arch. Oto-Rhino-Laryngol..

[50] Pérez Camargo D.A., Allende Pérez S.R., Verastegui Avilés E., Rivera Franco M.M., Meneses García A., Herrera Gómez Á., Urbalejo Ceniceros V.I. (2017). Assessment and Impact of Phase Angle and Sarcopenia in Palliative Cancer Patients. Nutr. Cancer.

[51] Hui D., Moore J., Park M., Liu D., Bruera E. (2019). Phase Angle and the Diagnosis of Impending Death in Patients with Advanced Cancer: Preliminary Findings. Oncol..

[52] Arab A., Karimi E., Vingrys K., Shirani F. (2021). Is phase angle a valuable prognostic tool in cancer patients’ survival? A systematic review and meta-analysis of available literature. Clin. Nutr. Edinb. Scotl..

[53] Norman K., Stobäus N., Pirlich M., Bosy-Westphal A. (2012). Bioelectrical phase angle and impedance vector analysis--clinical relevance and applicability of impedance parameters. Clin. Nutr. Edinb. Scotl..

[54] Mueller T.C., Reik L., Prokopchuk O., Friess H., Martignoni M.E. (2020). Measurement of body mass by bioelectrical impedance analysis and computed tomography in cancer patients with malnutrition—A cross-sectional observational study. Medicine.

[55] Katsura N., Yamashita M., Ishihara T. (2021). Extracellular water to total body water ratio may mediate the association between phase angle and mortality in patients with cancer cachexia: A single-center, retrospective study. Clin. Nutr. ESPEN.

[56] Galli A., Colombo M., Carrara G., Lira Luce F., Paesano P.L., Giordano L., Bondi S., Tulli M., Mirabile A., De Cobelli F. (2020). Low skeletal muscle mass as predictor of postoperative complications and decreased overall survival in locally advanced head and neck squamous cell carcinoma: The role of ultrasound of rectus femoris muscle. Eur Arch. Oto-Rhino-Laryngol.

[57] Bril S.I., Pezier T.F., Tijink B.M., Janssen L.M., Braunius W.W., de Bree R. (2019). Preoperative low skeletal muscle mass as a risk factor for pharyngocutaneous fistula and decreased overall survival in patients undergoing total laryngectomy. Head Neck.

[58] Formenti P., Coppola S., Umbrello M., Froio S., Cacioppola A., De Giorgis V., Galanti V., Lusardi A.C., Ferrari E., Noè D. (2021). Time course of the Bioelectrical Impedance Vector Analysis and muscular ultrasound in critically ill patients. J. Crit. Care.

[59] Souza N.C., Avesani C.M., Prado C.M., Martucci R.B., Rodrigues V.D., de Pinho N.B., Heymsfield S.B., Gonzalez M.C. (2021). Phase angle as a marker for muscle abnormalities and function in patients with colorectal cancer. Clin. Nutr..

[60] da Silva Passos L.B., Macedo T.A.A., De-Souza D.A. (2021). Nutritional state assessed by ultrasonography, but not by bioelectric impedance, predicts 28-day mortality in critically ill patients. Prospective cohort study. Clin. Nutr..

