# Supplementary material for: Rectus Femoris Muscle and Phase Angle as Prognostic Factor for 12-Month Mortality in a Longitudinal Cohort of Patients with Cancer (AnyVida Trial)
Source: Nutrients. 2023 Jan 19;15(3):522. doi: 10.3390/nu15030522 (PMC9919732; doi:10.3390/nu15030522)
Supplement: Supplementary file 1 [file nutrients-15-00522-s001.zip › nutrients-2091985-supplementary.pdf]

SUPPLEMENTARY MATERIAL

Table S1. Anthropometric parameters obtained in BIA. There is no predictive cut-off value for mortality in anthropometric parameters.

Parameter	Cut-off value	Sensitivity (%)	Specificity (%)	AUC	HR (CI; p-value)
BMI	24.3	32	62	0.477	0.97 (0.88-1.08; *p* = 0.57)
FFMI	19.7	12	78	0.458	0.91 (0.76-1.09; *p* = 0.32)
ASMM	24.4	12	81.25	0.426	0.94 (0.86-1.04; *p* = 0.24)
SMI	7.3	88	21.88	0.507	0.96 (0.75-1.24; *p* = 0.77)

Abbreviations. HR: Hazar Ratio; CI: confidence interval.

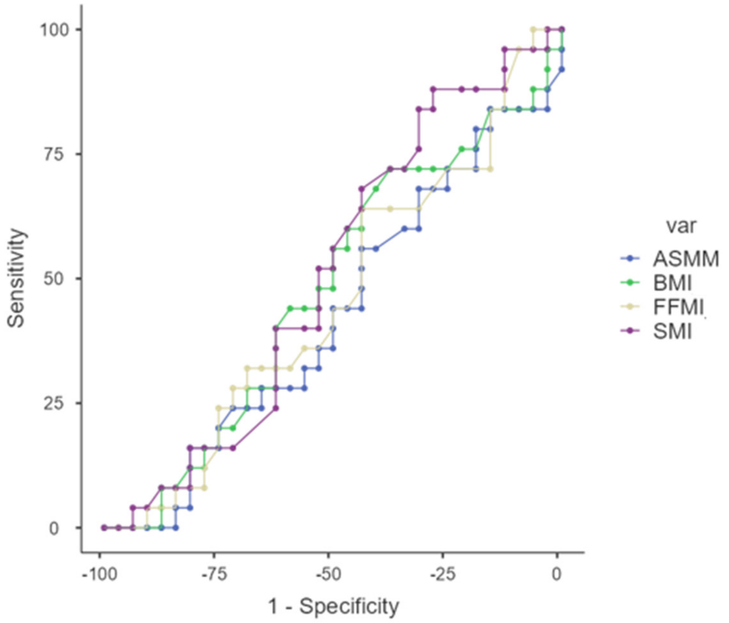

Figure S1. ROC Curve.

Area under the ROC curves (AUC) for predicting the survival at 12-months in cancer patients. ASMM, BMI, FFMI and SMI. Overall patients. ROC: receiver operating characteristic. **Abbreviations.** ASSM: appendicular skeletal muscle mass; BMI: body mass index, FFMI: fat free mass and SMI: skeletal muscle mass index. To interpret the color references in this figure legend, please see the Web version of this article. **Source**: own elaboration.
